# A branched TAT cell-penetrating peptide as a novel delivery carrier for the efficient gene transfection

**DOI:** 10.1186/s40824-016-0076-0

**Published:** 2016-09-07

**Authors:** Chanuk Jeong, Jisang Yoo, DaeYong Lee, Yeu-Chun Kim

**Affiliations:** Department of Chemical and Biomolecular Engineering, Korea Advanced Institute of Science and Technology, 291 Daehak-ro, Yuseong-gu, Daejeon 305-701 Republic of Korea

**Keywords:** Cell penetrating peptides, Gene delivery, Transfection, TAT, Branched TAT

## Abstract

**Background:**

Cell penetrating peptides (CPPs) as one class of non-viral vectors, have been widely explored as a delivery tool due to their cell-penetrating capability with low cytotoxicity. However, CPPs have reported to have low gene transfection efficiency mainly due to the fact that DNA is larger than other biomolecules. On the other hand, the conventional linear CPPs are unstable for constructing the DNA complexes with it. Thus, here we designed a branched CPP using disulfide bridges based on the linear TAT peptide, to enhance the gene delivery efficiency in a better way.

**Results:**

The branched TAT (BTAT) was synthesized by the DMSO oxidation method and showed high-molecular-weight about 294 kDa. The resulting BTAT was complexed with plasmid green fluorescence protein (pGFP) gene at various N/P ratios. The gene transfection efficiency was assessed on HeLa cells after treating with BTAT/pGFP complexes, showed high gene transfection efficiency as conformed by flowcytometry followed by confocal laser scanning microscopy (CLSM) visualization.

**Conclusion:**

The novel BTAT/pGFP complex exhibited significantly higher stability and redox cleavability by reducing agent. In addition, BTAT showed higher transfection efficiency approximately 40-fold than those of the TAT and mTAT complexes. Our primary experiments demonstrated the potential of BTAT as a suitable candidate for gene delivery and it could be applied for various types of gene delivery platforms.

## Background

Gene therapy is a powerful tool with the potential to inhibit the deleterious effects of malicious genes by inserting corrected/normal genes into the genome to treat the disease. Precisely, it could treat the disease by delivering specific nucleic acid into the target cells instead of drugs [1]. The concept of the gene therapy has been well known since 1970s [2] and it showed improved therapeutic effects in the various genetic diseases [3–6]. A variety of experimental results showed the benefits of gene therapy and further studies have been investigated to enhance the therapeutic effects by overcoming biological barriers such as immune response, high cellular toxicity and low transfection efficiency. To improve gene delivery capability with low cytotoxicity, various non-viral delivery vectors have been used such as liposome, cell-penetrating peptide (CPP) and cationic polymers [7–9]. Among the non-viral gene delivery tools, peptide-based vehicles have been widely used due to their biocompatibility and biodegradability [10–15].

Cell-penetrating peptides (CPPs) are representative peptide-based carriers and composed of short amino acid sequences less than 40. The CPPs possess positive charge because it consists of positively charged amino acids such as lysine and arginine. Being cationic in nature, CPPs could easily get inside the cells via various endocytosis mechanisms mediated by clathrin, and clathrin-independent endocytosis etc. [16]. Owing to these unique properties, the CPPs could interact with the negatively charged cellular membrane and enter the cells by means of their cell permeable characteristics without any cytotoxicty [16]. For over 20 years, CPP based gene delivery has been investigated [17–23] to enhance the transfection efficiency and introduce the targeting capability to the CPP/DNA complexes. The first CPPs were truncated from the transduction domain of the HIV-virus, TAT (48–60), since then it has been widely studied [24].

Although natural CPPs can penetrate into the cells without toxicity, transfection efficiency is too low to achieve expected therapeutic effects due to their low-molecular-weight, unstable linear structure and weak gene condensation capability. To overcome these drawbacks, various CPP modifications have been studied by conjugating with different chemical moieties. The branched structures are more advantageous than linear molecules to deliver biomolecules into cells [25]. To construct a branched structure, linear molecules must be linked with each other. As one method for linking, the disulfide bond is a simple and useful bridge for peptide modification because disulfide bridge can be easily linked by an oxidation method. In addition, the disulfide link is sensitively cleaved by reducing agents such as glutathione (GSH) which is overproduced in cytoplasm of cancer cells compared with normal cells [26]. Therefore, the branched peptide linked by a disulfide bond with each other would be degraded in cancer cells under reducing conditions.

**Fig. 1:**
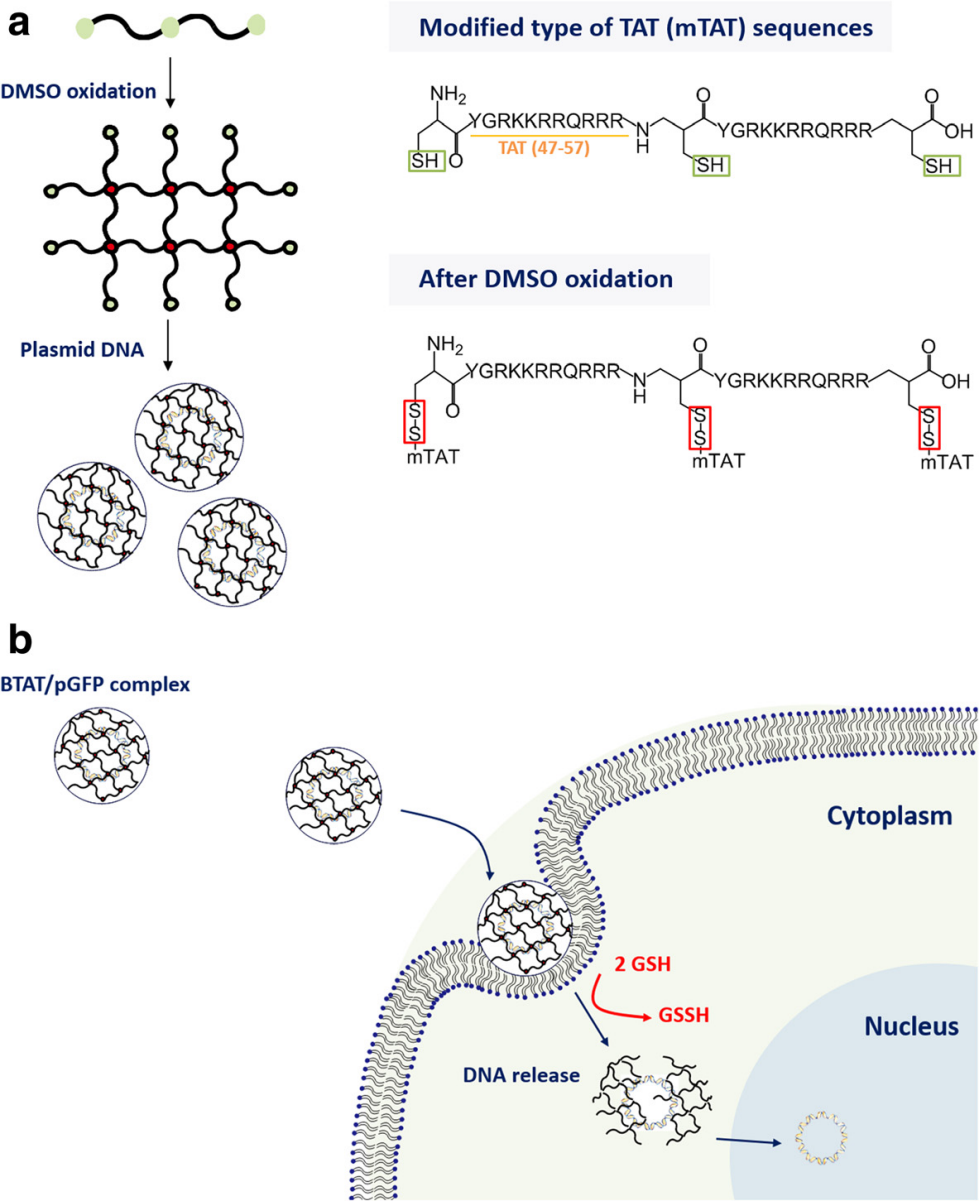
Synthesis and schematic illustration of the BTAT/pGFP complex delivery for the gene transfection

Herein, we designed the branched TAT (BTAT) using the modified type of TAT (mTAT, Cys-TAT-Cys-TAT-Cys) which contains cysteine sequences to construct a disulfide bonds. The thiol groups of the cysteines were linked with each other in mild oxidation conditions through dimethyl sulfoxide (DMSO). In the mTAT sequences, cysteines were positioned in the middle of the total sequence as well as the end site. Consequently, we hypothesize that disulfide bonds will be formed in horizontal and vertical directions as shown in Fig. 1a. The negatively charged pGFP could easily be complexed with positively charged branched TAT through electrostatic interactions, enabling better cellular permeability by means of CPP’s cell-permeable characteristics. The branched structures would be destroyed under reducing conditions in the cytoplasm of cancer cells, allowing the pGFP to be released as shown in Fig. 1b.

## Methods

### Materials

Dulbecco’s Modified Eagle’s Medium (DMEM), Fetal Bovine Serum (FBS), Antibiotic Antimycotic Solution (AAS), Polyethyleneimine (PEI, 25 kDa), Dimethyl Sulfoxide (DMSO) were purchased from Sigma-Aldrich (MO, USA). The Plasmids green fluorescence protein (pGFP, 5715 bp) gene was provided from KRIBB (Daejeon, Korea). The modified TAT (CYGRKKRRQRRRCYGRKKRRQRRRC) and TAT (YGRKKRRQRRR) were purchased from Peptron Co. Ltd (Daejeon, Korea). The HeLa (human cervical carcinoma, ATCC CCL-2) cell line was grown in DMEM supplemented with 10 % v/v FBS and 1 % v/v AAS. The cells were incubated in the CO_2_ incubator with 5 % CO_2_ at 37 °C for 1 day. The pGFP gene was extracted from *E. coli* using plasmid DNA extraction kit (HiSpeed Plasmid Maxi Kit, QIAGEN, Germany). According to the Maxi kit protocol, pGFP gene was obtained with high purity >1.8.

### Availability of data and materials

1. Plasmids green fluorescence protein (pGFP, 5715 bp) gene
  The pGFP gene was provided by KRIBB (Daejeon, Korea) and it is not a raw material. From plRES2-EGFP, take EGFP by PCR and clone the vector with using only Nhel at pcDNA 3.1/zeo (+).
2. HeLa cell line
  The HeLa (human cervical cancer, ATCC CCL-2) cell was purchased from the Korean Collection for Type Culture (KCTC) (Daejeon, Korea).

### The Branched TAT (BTAT) peptide synthesis

To synthesize the branched TAT (BTAT), mTAT (20 mg) was dissolved in PBS (pH 7.4) and 20 % v/v DMSO solvent. For the oxidation reactions of thiol groups of cysteines, mTAT solvent was stirred at the room temperature for overnight. The BTAT was diluted in the 10 mM HEPES buffer (pH 7.4) to avoid the extra reaction. The free mTAT and DMSO were removed by dialysis (MWCO 10000) for 1 day against distilled water. The purified BTAT was then obtained by lyophilization. BTAT was dissolved in distilled water at the concentrations of 0.15, 0.25, 0.5, 0.75 and 1 mg/mL. The absolute-molecular-weight was then measured by static light scattering (SLS) method using a Zetasizer (Zetasizer-Nano ZS90, Malvern Instruments, UK). The peptide structures of TAT, mTAT and BTAT were determined by a CD spectrometer (Jasco-815, Jasco, Japan).

### Formation of pGFP complexes

The TAT/pGFP, mTAT/pGFP, BTAT/pGFP and PEI/pGFP complexes were prepared by mixing with a pGFP (1 μg) at the different N/P ratios and incubated for 30 min at RT. The N/P ratio is calculated as follows: At N/P =1, BTAT (0.64 μg)/pGFP (1 μg).

### Gel retardation assay

To confirm the gene condensation capability of the BTAT, agarose gel retardation assay was conducted. All of the samples were prepared by mixing pGFP (0.5 μg) with the TAT, mTAT, BTAT and PEI at the various N/P ratios (0.3–8). After 30 min of incubation, each sample was dyed with Dyne Loading STAR (DYNE BIO, Korea) at 5:1 % v/v ratio. Agarose gel was prepared by dissolving the agarose (0.4 mg) in 40 mL of 1X Tris-acetate-EDTA (TAE) buffer. Each pGFP complex was loaded into the well of an agarose gel and electrophoresed for 20 min.

To determination of the redox cleavability of BTAT/pGFP complex, Glutathione (GSH) was added to the complex solution at a final concentration of 10 mM and incubated at 37 °C for 4 h.

### Size and zeta potential measurement

The pGFP (4 μg) was mixed with TAT, mTAT, BTAT and PEI at the various N/P ratios. After 30 min incubation, distilled water was added to each sample to the total volume of 800 μL. Sizes and zeta potentials were measured by dynamic light scattering (DLS, Zetasizer-Nano ZS90, Malvern Instruments, UK).

### Morphology analysis of BTAT/pGFP complex

The morphology of complex was determined by transmission electron microscopy (TEM, Tecnai F20 200 kV microscope, Philips). The BTAT/pGFP complexes were dropped on the carbon coated mesh grid (200-mesh) for 2 h.

### Cell viability test

The HeLa cells were seeded into the 96-well plates at 1 × 10^4^ cells/well and incubated for 24 h. After 1 day, Opti-MEM containing pGFP (0.25 μg) complexes at the various N/P ratios were treated into the each well. After incubation for 24 h, MTT (5 mg/ml in PBS) solution was added to the each well and incubated for 3 h. Thereafter, DMSO (100 μL) was added and the absorbance was measured at 590 nm wavelength through microplate reader (Multiskan™ Go Microplate Spectrophotometer, Thermo Co., U.S.A).

### Transfection efficiency of pGFP complex

The HeLa cells were seeded into the 24-well plates at 4 × 10^4^ cells/well and incubated for 24 h at 37 °C. The Hela cells were treated with Opti-MEM (450 μL) containing pGFP (1 μg) with TAT, mTAT and BTAT at an N/P ratio 4 and incubated for 4 h. The cells washed with phosphate-buffered saline (PBS) and fresh DMEM supplemented with 10 % v/v FBS and 1 % v/v AAS was added. The cells were then incubated for 2 days and medium were removed. After washing with PBS three times, the cells were detached by trypsin. The transfection efficiencies were measured by flow cytometry (BD FACSCalibur, BD Biosciences, USA).

For qualitative analysis, the cell nuclei were stained with DAPI for 10 min and fixed by 4 % paraformaldehyde. The cells were then washed three times with PBS and mounted on the slide glass. The cellular images were then analyzed and captured by the confocal laser scanning microscopy (CLSM, C2+, Nikon, Japan).

## Results and discussion

### Molecular weight and secondary structure of the branched TAT (BTAT)

**Fig. 2:**
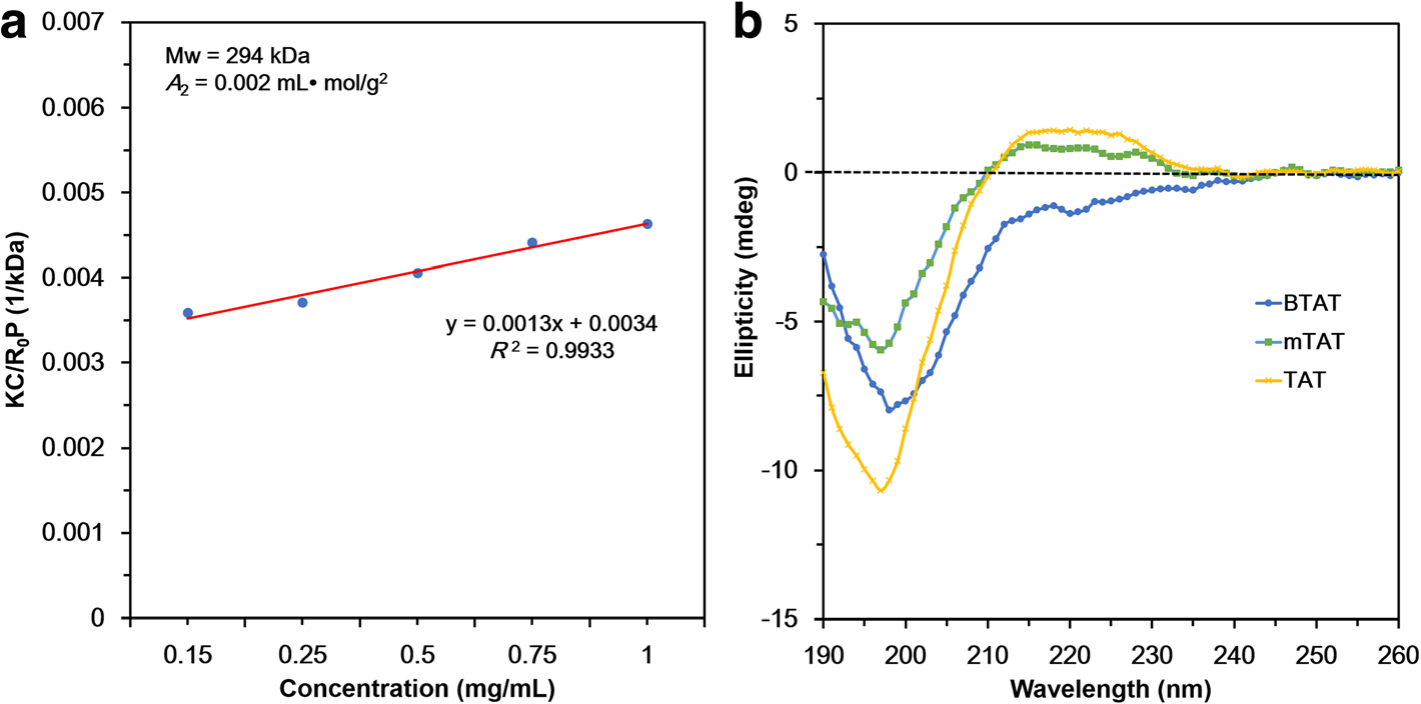
Synthesis and characterization of the BTAT. **a** The absolute molecular weight of the branched TAT (**b**) The CD spectra determination of the TAT, modified TAT and branched TAT

The branched TAT (BTAT) was synthesized via disulfide bridges using modified type of TAT containing the cysteine residues. After DMSO oxidation, the mTAT solution was changed to clear gel form, indicative of BTAT formation. It could be of mTATs in solution linked with each other by disulfide bonds, allowing structure from linear to branched one, resulting in gel formation of BTAT Table 1. After DMSO oxidation, the absolute-molecular-weight was measured by static light scattering (SLS) using the various concentrations of BTAT. As shown in Fig. 2a, the absolute-molecular-weight of BTAT was calculated approximately 294 kDa. The molecular weight increased from mTAT (3.4 kDa) up to 86-fold, indicating that the branched structure was successfully constructed by disulfide linkages.

**Table 1:** Amino acid sequences, molecular weight and number of amino acids of CPPs

CPPs	Amino acid sequences	Molecular weight (Da)	Number of amino acids
TAT	YGRKKRRQRRR	1560	11
mTAT	CYGRKKRRQRRRCYGRKKRRQRRRC	3409	25

The secondary structure of the TAT, mTAT and BTAT was determined by using the CD spectra. The secondary structure of the TAT peptide was known as random coil [27]. As shown in Fig. 2b, CD spectra showed a random coil structure of TAT. Similarly, mTAT and BTAT also showed the identical structures, meaning that disulfide linkages did not affect the secondary structures.

### Gel retardation assay

**Fig. 3:**
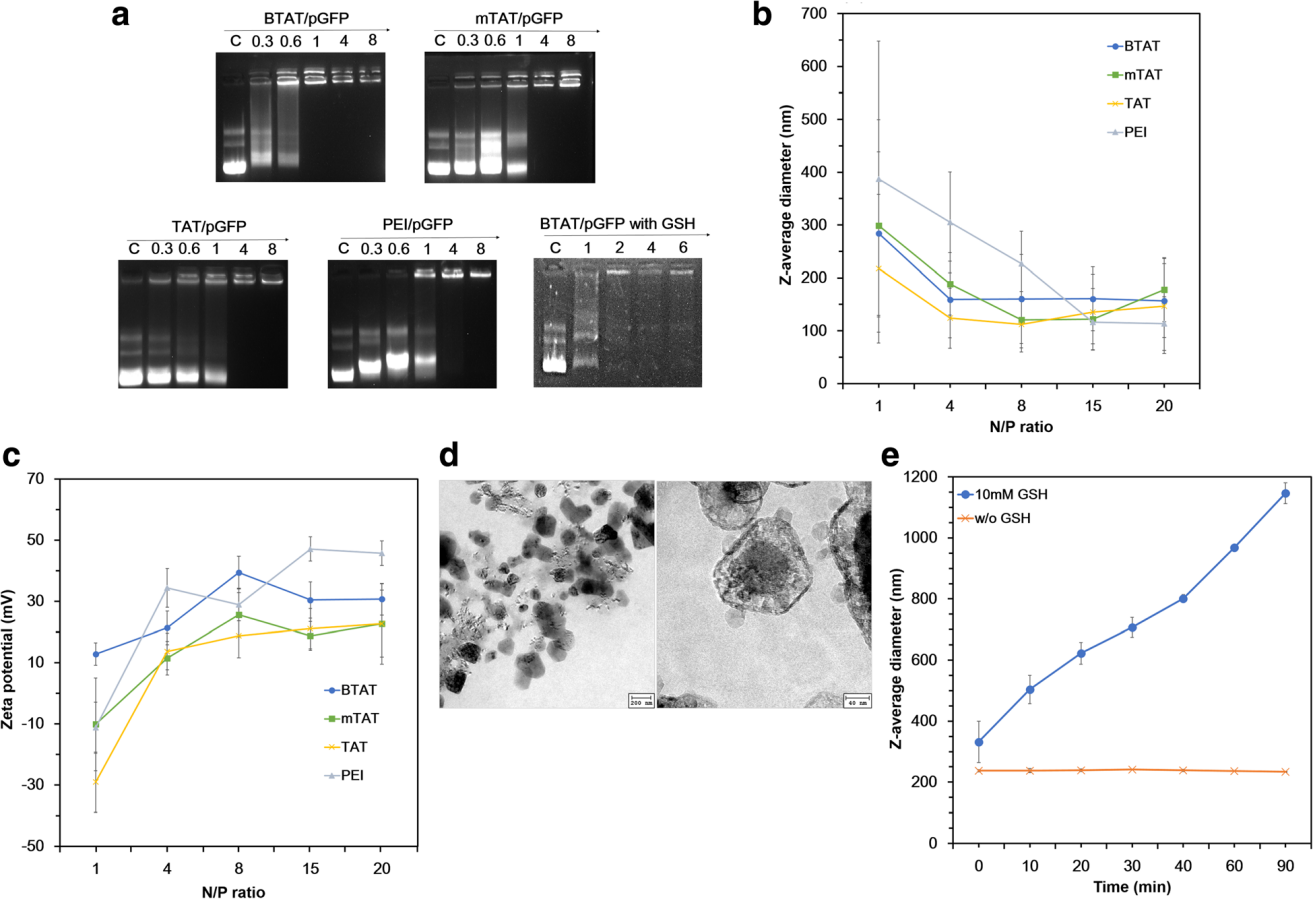
**a** The agarose gel retardation assays and **b** Particle sizes and **c** zeta-potential values of the CPP/pGFP complexes at the various N/P ratios. **d** TEM images of the BTAT/pGFP complex. **e** Size change of the BTAT/pGFP complex in water after 10 mM GSH treatment

The gene condensation capability of each complex was analyzed by the gel retardation assay at the various N/P ratios, as shown in Fig. 3a. In case of the BTAT complex, the improved binding ability was observed compared with TAT, mTAT and PEI complexes. The BTAT/pGFP complex was completely retarded at an N/P ratio 1, while TAT and mTAT were retarded at an N/P ratio 4. These data suggest that the branched TAT was able to condense the pGFP at the low N/P ratios. These enhanced gene condensation capability could be well explained based on the structure of BTAT. Being more branched, it would allow the pGFP to well interact with the positively charged random coils of BTAT, enabling improved interaction within the structures than the other forms.

### Particle size and zeta-potential

For effective gene delivery, the CPP/pGFP complexes should possess small particle size and positive charge. As shown in Fig. 3b, the size determinations of the complexes were investigated at the various N/P ratios ranging from 1 to 20. Particle sizes of all complexes reduced with growing the N/P ratios due to increase of gene condensation capability. The BTAT could condense pGFP with a diameter of approximately 300 nm for an N/P raito 1 whereas < 200 nm was obtained for the N/P ratios from 4 to 20. Similarly, TAT and modified TAT showed the identical trends at the N/P ratios ranging from 1 to 20. On the other hand, the PEI which is commercially used transfection agent exhibited a large particle than the CPP complexes at an N/P ratio 1 with size of > 300 nm.

The zeta-potential value of each CPP/pGFP complex was measured at the N/P ratios ranging from 1 to 20. As shown in Fig. 3c, all of the samples showed the similar trends. The zeta potential values were increased with increasing N/P ratios as it changed from negative to positive charge except for BTAT. Only the BTAT/pGFP complex exhibited a positive charge at an N/P ratio 1. According to the previous reports, the positive charge of complex is essential for penetration into cells by electrostatic interaction with negatively charged cellular membrane [16]. Based on these results, although the molecular weight was much higher than that of PEI, the BTAT/pGFP complex had reasonable particle size to penetrate into cells.

### Morphology analysis of BTAT/pGFP complex

To confirm the morphology of the BTAT/pGFP complex, the BTAT/pGFP complex was prepared at an N/P ratio 4. As shown in Fig. 3d, the TEM images showed a spherical nano-structure for BTAT complexes. Figure 3d clearly shows that the DNA was mostly encapsulated in BTAT structure and the BTAT/pGFP particle size was about 200 nm at an N/P ratio 4. The branched structure and condensed DNA was well formulated in BTAT/pGFP as visualized by the TEM image (Fig. 3d).

### Redox cleavability of BTAT complex

To demonstrate the cleavability of the BTAT linked with disulfide bonds, the BTAT/pGFP complexes were incubated in 10 mM GSH solution at the various N/P ratios. The gel retardation assay was then conducted to confirm whether the DNA could release or not under reducing environments. As shown in Fig. 3a, the BTAT/pGFP complex was degraded and the pGFP band moved to bottom of agarose gel at the N/P ratios ranging from 1 to 6.

In addition, the particle size changes under reductive conditions were investigated in presence and absence of GSH. In the presence of GSH, the particle size significantly increased up to 1000 nm (Fig. 3e). On the other hand, the average size of the BTAT/pGFP complex was not changed when dissolved in the distilled water without 10 mM GSH. These results showed that the BTAT composed of disulfide bonds possesses redox cleavability in reductive conditions and this property could be applied for cancer cell targeting system.

### Cell viability test

**Fig. 4:**
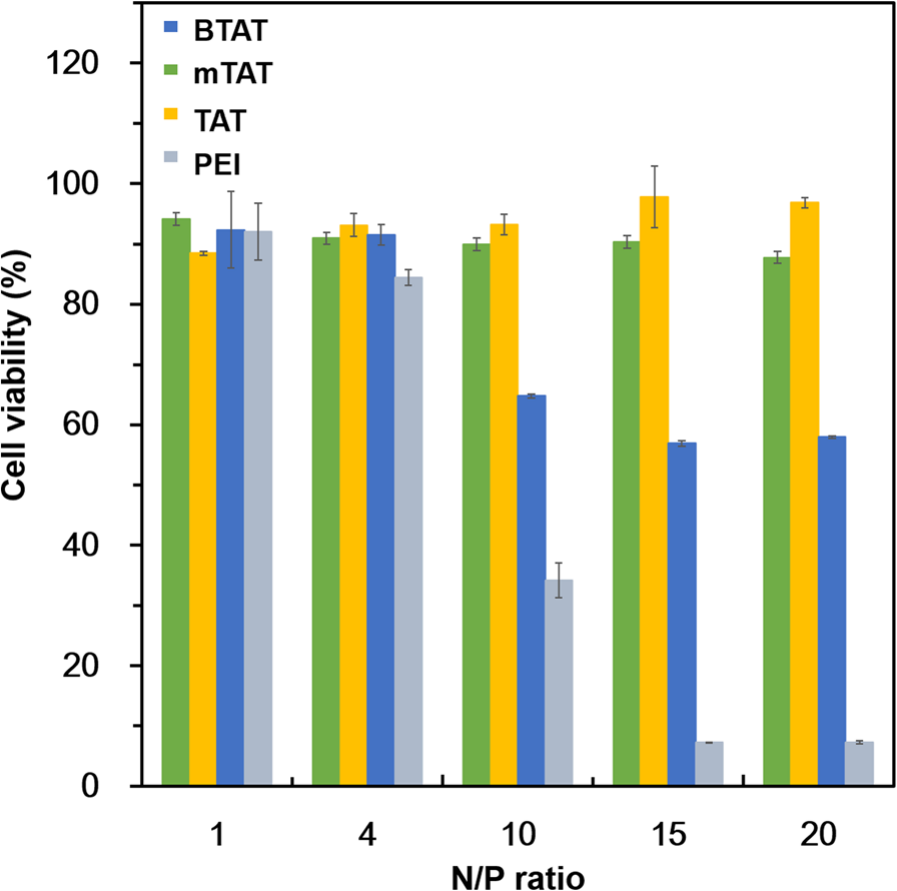
Cell viability test. Dose-dependent cytotoxicities of CPP/pGFP complexes in the HeLa cells

The cell viability of the CPP/pGFP complexes was determined by MTT assay in the HeLa cells. As shown in Fig. 4, all of the CPP/pGFP complexes were shown more than 80 % cell viability at an N/P ratio of 4, meaning that all of the complexes were cytocompatible at the lower N/P ratios. The Poly (ethylenimine) (PEI) was used as a positive control. PEI is a polycation commonly used as gene transfection agent due to the high transfection efficiency and its easy usability. However, the PEI based gene delivery systems often induce high cytotoxicity problems. In our study, PEI observed to be cytotoxic as indicated in a gray bar in the Fig. 4. However the PEI/pGFP complexes were found to be cytocompatible at an N/P ratio 4, while it showed high cytotoxicity at an N/P ratio of 10. On the other hand, the BTAT/pGFP complex exhibited low toxicity than those of PEI at the identical N/P ratios. Despite the high concentrations of BTAT, the BTAT/pGFP complex could show more than 50 % of the cell viability, while the PEI/pGFP complexes decreased the cell viability approximately 10 % at the N/P ratios of 15 and 20, indicating the high cell viability of the BTAT/pGFP complex was contributed by the effective cleavage of the disulfide bonds in the reductive environments.

### Transfection efficiency of pGFP complex

**Fig. 5:**
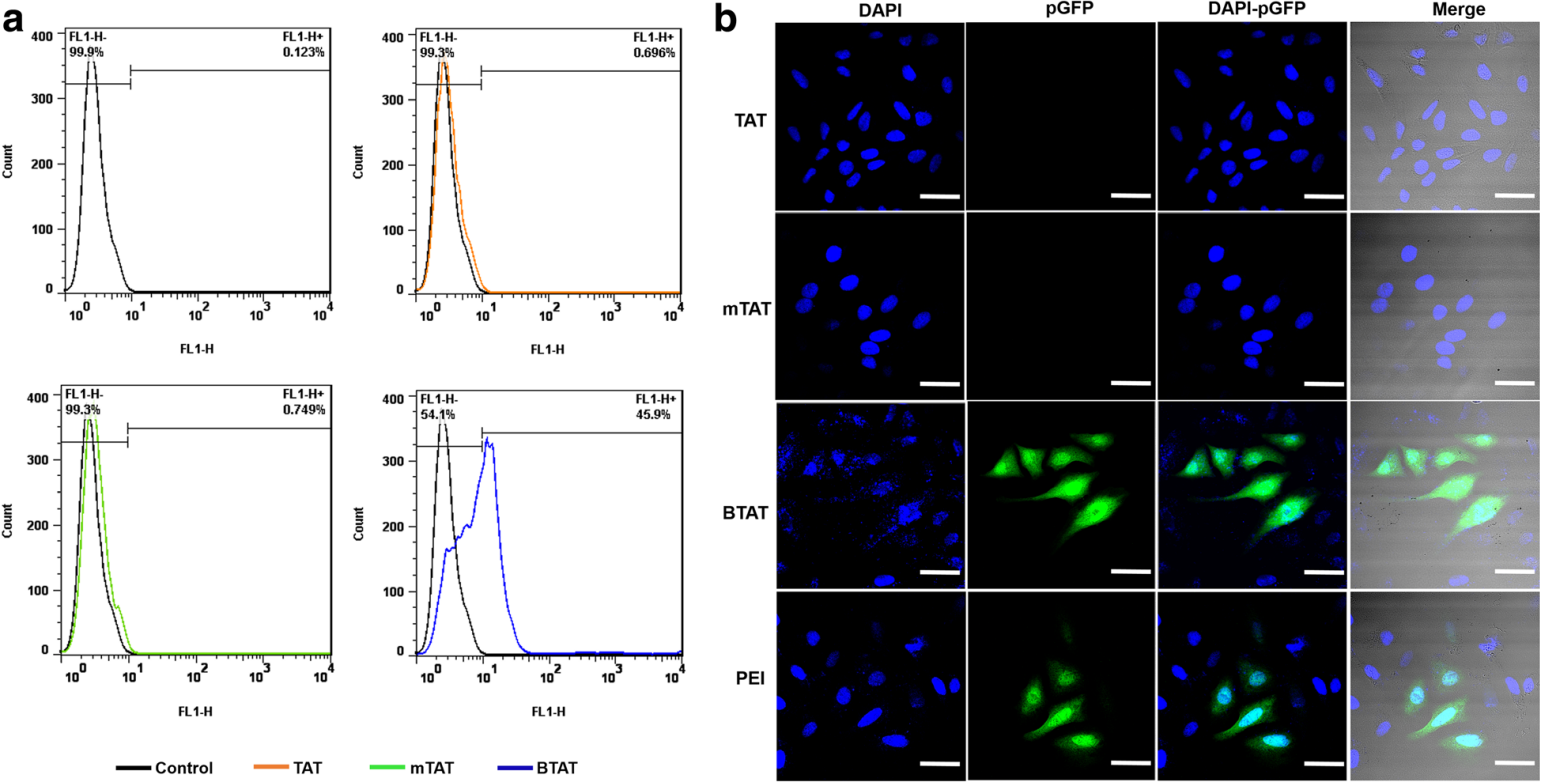
Transfection efficiency of pGFP complex in HeLa cells. **a** Quantification of the transfection efficiency using flow cytometry (**b**) Confocal laser scanning microscopy images of the expression of the green fluorescence protein. The images obtained at a 200x magnification. The scale bars represent 50 μm

The proportion of the transfected cells was shown in Fig. 5a. The HeLa cells were transfected by TAT/pGFP, mTAT/pGFP and BTAT/pGFP at an N/P ratio 4 and the proportion of the transfection was approximately 0.7 ± 0.26 %, 0.75 ± 0.25 % and 45.9 ± 1.87 %, respectively. It showed that the BTAT has superior transfection efficacy compared with conventional CPPs and PEI. This could be explained based on their branched networking and high positive charges to condense the pGFP complexes allowing an effective penetration into the cells.

To visualize the transfected cells, confocal laser scanning microscope (CSLM) analysis was conducted. TAT, mTAT and BTAT were complexed with pGFP at an N/P ratio 4. The PEI/pGFP complex was prepared at an N/P ratio 5 as a positive control. As shown in Fig. 5b, the green fluorescence from HeLa cells indicating high transfection efficiency of BTAT, whereas negligible fluorescence was observed with the TAT/pGFP and mTAT/pGFP complexes. Though, PEI showed green fluorescence, the BTAT showed significantly higher mean fluorescence intensity than all other samples.

## Conclusion

In our study, the BTAT has been made successfully to demonstrate its potential as gene delivery carrier with improved cytocompatibility than the well explored PEI. The mTAT peptide was conjugated with each other by disulfide linkage in horizontal and vertical direction. Owing to the high charge density of BTAT, it formed a stable complex with pGFP. The novel BTAT/pGFP complex showed higher transfection efficiency of approximately 40-fold than those of the TAT and mTAT complexes. In addition, despite the high positive charge of BTAT, the BTAT/pGFP complex showed high cell viability as the disulfide links were degraded in the cytoplasm. As we expected, the positively charged BTAT could penetrate the cells efficiently, with high gene transfection efficiency. Therefore, the BTAT may act a powerful tool for gene delivery and has great potential to apply to any other types of the gene delivery platforms.

## Abbreviations

AAS: Antibiotic antimycotic solution
BTAT: Branched TAT
CPPs: Cell penetrating peptides
DMEM: Dulbecco’s Modified Eagle’s Medium
DMSO: Dimethyl sulfoxide
FACS: Fluorescence-activated cell sorting
FBS: Fetal bovine serum
GSH: Glutathione
HEPES: 2-[4-(2-hydroxyethyl) piperazin-1-yl] ethanesulfonic acid
mTAT: Modified TAT
PEI: Poly (ethyleneimine), CLSM, confocal laser scanning microscope
pGFP: Plasmid green fluorescence protein
TEM: Transmission electron microscopy
